# Phylogeography and Genetic Structuring of European Nine-Spined Sticklebacks (*Pungitius pungitius*)—Mitochondrial DNA Evidence

**DOI:** 10.1371/journal.pone.0019476

**Published:** 2011-05-11

**Authors:** Amber G. F. Teacher, Takahito Shikano, Marika E. Karjalainen, Juha Merilä

**Affiliations:** Ecological Genetics Research Unit, Department of Biosciences, University of Helsinki, Helsinki, Finland; Lund University, Sweden

## Abstract

As a consequence of colonisation from different glacial refugia, many northern European taxa are split into distinct western and eastern lineages. However, as for the nine-spined stickleback (*Pungitius pungitius*), the exact location of the contact zone between lineages often remains poorly known. We assessed the genetic differentiation and diversity in the nine-spined stickleback within Europe using 1037 base pairs of cytochrome b sequence for 320 individuals from 57 locations, including pond, lake, river, and coastal habitats. Our main aims were (i) to locate the contact zone between the previously recognized western and eastern lineages, (ii) investigate latitudinal patterns in genetic diversity, (iii) compare genetic diversity among different habitat types, and (iv) date the known split between eastern and western lineages. The data revealed the split between eastern and western to be located across the Danish Straits and roughly following the Norway/Sweden border to the North. Reference sites from Canada form their own clades, and one of the Canadian sites was found to have a haplotype common to the Eastern European lineage, possibly representing an ancestral polymorphism. The split between the two European clades was dated to approximately 1.48 million years ago (Mya), and between Canada and Europe to approximately 1.62 Mya. After controlling for habitat effects, nucleotide (but not haplotype) diversity across populations decreased with increasing latitude. Coastal populations showed significantly higher haplotype diversity (but not nucleotide diversity) than pond populations, but there were no detectable differences in haplotype diversity among different freshwater habitat types (viz. river, lake and pond populations), or between coastal and lake/river populations. Sequences were found to cluster according to their geographic proximity, rather than by habitat type, and all habitat types were found within each major clade, implying that colonisation and adaptation between the coastal and freshwater environments in different regions must have occurred in parallel.

## Introduction

Towards the end of the Pleistocene (approximately 10 k years ago), the ice sheets that had covered most of Northern Europe began to retreat, and temperate species began to re-colonise the newly habitable Northern regions [1], [2]. The origins of the current genetic distributions of European terrestrial species have been well studied with respect to the Pleistocene glaciations, revealing genetic signatures corresponding to refugial origins and recolonisation routes (e.g. [3], [4]). Aquatic species have also been found to show deep phylogenetic splits within Europe, usually corresponding to lineages originating from southern and central European refugia (e.g. Brown trout [5], [6]; Bullhead [7]; Grayling [8]; Perch [9]).

One of the youngest marine regions in Northern Europe is the Baltic Sea; a large and somewhat isolated brackish water inlet. The Baltic Ice Lake, now the Baltic Sea, formed as the glaciers retreated [10]. Colonisation of the Baltic Sea could in theory have taken place from the West, following a marine connection appearing between the Baltic Ice Lake and the Atlantic, or from the East via river drainages from glacial refugia - these existed about 5k years before the Atlantic connection opened [10]. The colonisation routes of aquatic species into Fennoscandia vary (reviewed in [11]), but two major clades (eastern and western) are often recognised. For example, mitochondrial DNA and microsatellites both show that the European grayling (*Thymallus thymallus*) fall into two major clades within Fennoscandia; an eastern lineage that occurs in Finland, Russia and Sweden, and a western lineage that occurs in Sweden and Denmark [8], [12]. This finding is mirrored by mitochondrial DNA analyses of nine-spined stickleback (*Pungitius pungitius*), revealing two main lineages within Fennoscandia; a low diversity Eastern lineage found in Finland, Russia, Estonia and Sweden, and a higher diversity Western lineage that is found in Denmark, the UK, France, Belgium, and Norway [13]. One factor that may help to maintain the split between these Eastern and Western lineages is the presence of a semi-permeable barrier to migration – the Danish Straits. The Danish Straits are three narrow channels which currently connect the Baltic Sea to the North Sea, via the Kattegat and Skagerrak, and the effects of this semi-permeable barrier can be detected in marine species. For example, Atlantic salmon (*Salmo salar*) populations on the Swedish West coast differ from those close-by in the Southern Baltic, indicating no inward (into the Baltic), and little outward gene flow through the Danish straits [14]. The Danish straits currently mark a dividing line where the genetic diversity of populations markedly drops from the Atlantic to the Baltic among 29 marine species studied [15]. There is also a hypothesis of a marine and freshwater glacial refugium near the White Sea region. Marine populations from the White Sea sometimes appear to be genetically distinct from those in the Baltic Sea. For example, Atlantic salmon in the Baltic Sea basin appear to originate from a South-Eastern ice lake refugia and the Atlantic [14], [16], [17], whilst those in the White and Barents Sea may have been colonised from populations in Russia (e.g. the Komi Ice Lake in Northern Russia) together with some Atlantic immigrants [18], [19].

The process of colonisation is usually associated with a loss of genetic diversity towards the leading edge of the colonizing front, caused by founder effects and serial bottlenecks [20]. This often manifests itself as a decrease in genetic diversity towards the North of Europe, as the majority of postglacial European colonisation (at least for terrestrial species) is thought to have occurred from large Southern refugia [1]. In species that can live in both marine and freshwater habitats, there is potential for patterns of diversity to be complicated further. Theory predicts that marine fish populations will show higher levels of genetic diversity and a lower degree of differentiation due to high gene flow and large effective population sizes as compared to freshwater fish populations [21], [22]. In contrast, freshwater populations in lakes, ponds, and rivers are expected to show lower diversity and a higher degree of differentiation [23], [24]. These major differences between marine and freshwater populations have been shown to hold empirically in a wide range of species (e.g. 78 fish species [22]; 105 fish species [25]; 68 fish species [26]), including the three-spined [27] and nine-spined [13] sticklebacks. Adaptation to freshwater, and the colonisation of the inland freshwater habitats by three-spined stickleback seem to have occurred by multiple independent colonisation events from local marine ancestors (i.e. parallel evolution, as opposed to common ancestry), throughout most of the range of this species [27]–[29], though equivalent information is not yet available for the nine-spined stickleback.

**Figure 1:**
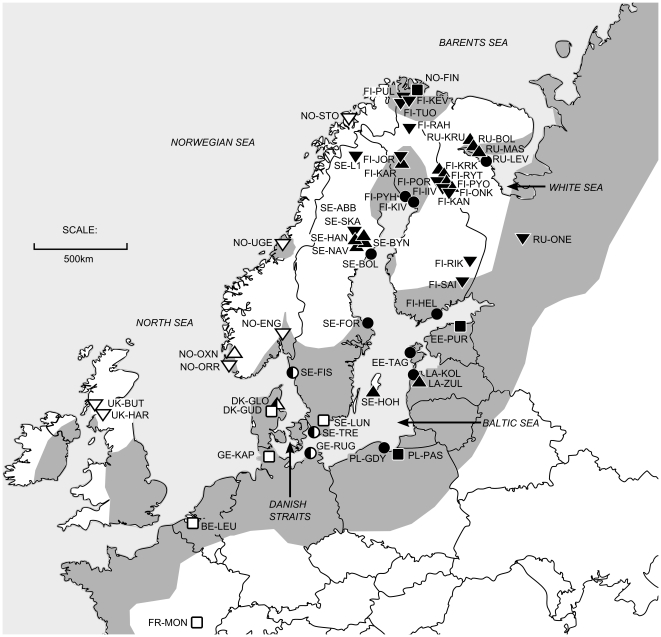
Map showing the sampling locations within Europe. Different marker shapes represent different habitat types (circle = sea, square = river, triangle = pond, inverted triangle = lake), whilst different colours represent the Eastern clade (black), and the Western clade (white). Markers with two colours show sites where haplotypes from multiple clades were found. In addition, there are two sites in Canada that are not shown – one is a pond site, and one is a lake site. Both sites form their own clade, however one sample from the pond site has haplotype E3, the most common Eastern haplotype. The distribution of the nine-spined stickleback is overlayed in grey (from Paepke [32]), and the major seas are labelled.

The main aim of this study was to use cytochrome *b* sequence analyses to expand on the current knowledge [13], [30] of the phylogeography of the nine-spined stickleback (*Pungitius pungitius*) - a small (ca. 4–6 cm) cold-adapted fish species which has lives in both coastal and freshwater habitats of the northern hemisphere ([31]; Fig. 1). In particular, we wished to address four specific issues. First, by building upon results from Shikano et al. [13], we aimed to narrow down the location of the division between the earlier recognized Eastern and Western lineages, and to see whether there is any overlap in their distributions. At present, the Western lineage has been identified from only a handful of localities, and large parts of Southern and South-western Fennoscandia have remained unstudied [13], [30]. We devised our sampling strategy to cover the existing gaps, particularly by the inclusion of samples from central and northern Norway, southern Sweden, and critically, 12 locations distributed between Denmark and Estonia (Fig. 1). Our second aim, again building upon results from Shikano et al. [13], was to assess the latitudinal (and longitudinal) patterns of genetic diversity within the two recognized clades that might have been caused by founder effects during recolonisation. Our sampling strategy was devised to improve existing sampling, and to cover the gaps in the latitudinal (and longitudinal) representation of populations sampled by Shikano et al. [13]. Our third main aim was to compare levels of genetic diversity among different habitat types (*viz*. pond, lake, river and sea populations) to see whether the evidence for reduced autosomal genetic diversity in freshwater populations [13] also holds in the case of mtDNA. Our fourth and final aim was to date the split between the two recognized European clades in order to provide a better understanding of the timeframe for the existing differentiation.

## Methods

### Ethics Statement

The samples used in this study were collected in accordance with the national legislation of the countries concerned.

### Sampling

The current distribution of nine-spined stickleback in Europe approximately follows the Northern coast-line of Europe down to France, including most of Fennoscandia and the UK/Republic of Ireland [32] (Fig. 1). We sampled 233 individuals from 41 locations, and combined our dataset with 87 sequences from 24 locations from a previously published paper [13], making a total of 320 sequences from 57 locations (see Fig. 1 and File S1 – two Canadian sites included as reference samples are not shown on the map). Many of the locations sampled in our dataset were newly sequenced for this study (n = 33), for some locations from Shikano et al. [13] we improved the sample size (n = 21), and for some locations we only used the previously published data (n = 3). In total, we had sequences for 17 pond, 20 lake, 8 river, and 12 coastal locations covering the area where the split between the Western and Eastern lineages was suspected to be found (c.f. [13]).

### Sequencing

Total DNA was extracted from ethanol-stored fin-clips with either a silica-fines/microtitre filtration plate method [33], or phenol-chloroform [34]. The primers for cytochrome *b* amplification were from Shikano et al. [13] - for each individual, two overlapping sequences were amplified with primer pairs L14724+PP14959 and PP14896+Cb6Thr, or if latter failed PP15469 was used instead of Cb6Thr. The polymerase chain reaction (PCR) mix was as follows: 1xPhire® Reaction Buffer (Finnzymes, Espoo, Finland), 0.2 mM of each dNTP (Finnzymes, Espoo, Finland), 3% of DMSO (Finnzymes, Espoo, Finland), 0.3 µl of Phire® Hot Start I DNA Polymerase (Finnzymes, Espoo, Finland) and 15 pmol of each primer, in a total reaction volume of 30 µl. The PCR program consisted of a preliminary denaturation step at 98°C for 30 s followed by 34 cycles of 98°C for 10 s, 60°C for 15 s, 72°C for 20 s and final extension at 72°C for 1 min. We ran 2 µl of each amplicon on 2% agarose gels (Bioline, Turku, Finland) to confirm amplification. PCR product purification and sequencing was performed by Macrogen Inc. (Korea) on an ABI 3730XL DNA Analyzer (Applied Biosystems). Sequence reads were manually checked, contigs assembled and sequences aligned using Geneious Pro 5.0.0 [35]. Aligned sequences were 1037 base pairs long, with no indels or ambiguities, and the haplotype sequences are available through GenBank (Accession numbers: JF798872-JF798929). These were combined with trimmed sequences from Shikano et al. [13].

### Phylogenetic trees

We used jModeltest v.0.1.1 [36], and the Akaike Information Criterion (AIC), to select the best-fit model of nucleotide substitution. The sequences were then collapsed to haplotypes using FaBox [37], and haplotypes were numbered to match the previous system of Shikano et al. [13]. A Bayesian consensus tree of the haplotypes was constructed using MrBayes v.3.1.2. [38], sampling every 100 generations for 7×10^6^ generations with four chains (average standard deviation of split frequencies <0.01). We excluded the first 25% of samples as burn-in, and coded the chosen model of nucleotide substitution. A published Japanese sample from the same species (GenBank accession number GU227782) was used as an outgroup to root the tree (c.f. [13]). In addition, a distance-based tree was constructed using DnaSP [39] to calculate N_ST_ between all population pairs, and using MEGA [40] to create a Neighbour Joining tree from the resulting distance matrix. One sample from Baffin Island in Canada which clustered with European samples was rerun for PCR and sequencing to verify that it was correct.

### Clade dating

Time to the most recent common ancestor (TMRCA) was estimated using an uncorrelated lognormal relaxed molecular clock model, implemented in Beast v1.5.4 [41]. To calibrate the rate of divergence, the brook stickleback (*Culaea inconstans*, Genbank accession number AB445125) and three-spined stickleback (*Gasterosteus aculeatus*, AB054361) were incorporated into the analysis. Due to the possible heterogeneity of evolutionary rates in different timescales [42], the analysis was performed in two steps following McCartney & Barret [43]: the first using outgroups and a small number of divergent ingroup sequences, and the second using no outgroups but all ingroup sequences. We included only three divergent haplotypes from three European and Canadian clades (E32, E70 and E86; see results) and a Japanese haplotype (GU227782) in the dataset containing the outgroups. In this analysis, the divergence time was set to 7.0 Mya for the split between brook and nine-spined sticklebacks [44], and 13.3 Mya between three-spined and nine-spined sticklebacks [45] with lognormal fossil priors. Two independent Markov chain Monte Carlo (MCMC) runs were conducted for 5×10^7^ generations under an HKY+I+G substitution model, assuming a Yule speciation process [46]. Parameters were logged every 5×10^3^ generations. After discarding the first 5×10^6^ generations as the burn-in, the TMRCA of the four haplotypes of nine-spined sticklebacks was estimated from the two MCMC chains using Tracer v1.5 [47]. These estimates were used as the calibration for a second analysis, which included all nine-spined stickleback samples but no other species. In this analysis, we assumed a normal TMRCA distribution, allowing divergence dates to vary symmetrically. Two independent MCMC runs were performed under an HKY+I+G substitution model, assuming a coalescent prior of constant population size [48]. Each MCMC chain was run for 5×10^7^ generations, sampling every 5×10^3^ generations. The mean and 95% highest posterior density (HPD) for the dates of divergence were summarized from the two MCMC chains using Tracer v1.5.

### Genetic diversity

Haplotype diversity was calculated globally, within the Eastern and Western clades, and for each population (excluding EE-TAG as there was only one sample for this population), using the formula 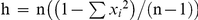, where *x_i_* is the haplotype frequency, and n is the sample size [49]. Mean nucleotide diversity was calculated for each population (except EE-TAG), using MEGA v.4 [40]. Outliers were checked and confirmed as true results. General linear models were implemented in R [50], where either haplotype diversity or nucleotide diversity was set as the dependent variable, the habitat type as a fixed factor, and latitude and longitude as covariates. Each term was dropped sequentially, and the models re-run. To look in more detail at the effect of different habitat types on haplotype and nucleotide diversity, differences between means of habitat type pairs were compared using a Tukey's post-hoc tests for multiple comparisons of means. All models were run for the whole dataset, only the freshwater samples (as high gene flow in coastal populations could affect the results), and within the Eastern and Western clades individually (excluding sites that contained individuals with haplotypes from both the Eastern and Western clades). Box plots showing the distribution of the nucleotide and haplotype diversities for each habitat type were produced in R [50]. Heterozygosity values from 12 microsatellite markers for 35 of the sites were obtained from Shikano et al. [13], and correlations between microsatellite heterozygosity and the mitochondrial DNA nucleotide and haplotype diversities obtained in this study (see File S1) were tested using two-sided Spearman's Rank Correlation Coefficients, implemented in R [50].

## Results

### Sequencing

The 1037 base pair cytochrome *b* sequences consisted of 93 haplotypes (overall haplotype diversity = 0.872), and 98 variable sites. Haplotypes E1–39 (n = 39) had previously been identified by Shikano et al. [13], whilst E40–E97 (n = 58) were newly identified in this study (File S1). As we have used a slightly shorter fragment (67 fewer base pairs) than the previous study, some haplotypes that previously differed now fall within the same group; thus the haplotypes previously identified as E3/E8/E16 are now identical (from now on simply referred to as E3), as are the haplotypes E28/E31/E33 (from now on referred to as E28). Hence, the numbering goes up to E97 despite there being only 93 haplotypes.

### Phylogenetic trees and clade-dating

**Figure 2:**
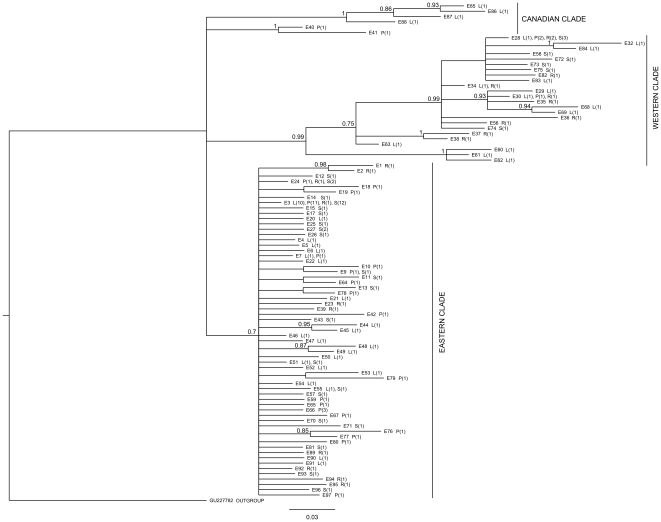
Bayesian phylogeny of 102 cytochrome *b* haplotypes, with main clades marked. Haplotype numbers are given (E1–106), followed by the site types represented (P = pond, L = lake, R = river, S = sea), and the number of such sites in brackets. Posterior probabilities >0.7 are indicated at the major nodes, and major clades are labelled. The outgroup is labelled by its accession number (GU227782), and is a previously published nine-spined stickleback sequence from Japan.

The best-fit model of nucleotide substitution, as determined by jModeltest, was the TIM2+I+G model with unequal base frequencies (freqA = 0.250, freqC = 0.308, freqG = 0.156, freqT = 0.287), unequal substitution rates (r_AC_ = 8.506, r_AG_ = 75.551, r_AT_ = 8.506, r_CG_ = 1.000, r_CT_ = 27.296, r_GT_ = 1.000, scaled to r_GT_), proportion of invariable sites = 0.670, and a gamma shape distribution = 0.910. The Bayesian tree revealed three main clades, namely Canadian, Eastern and Western European clades (Fig. 2). The Eastern clade members exhibit a low degree of divergence (but a high haplotype diversity: h = 0.997) and comprised of samples from Russia, Finland, Northern Norway, Sweden, Latvia, Estonia, Poland, Germany and Denmark. The Western clade had a higher degree of intraclade divergence (but a lower haplotype diversity of 0.852), and comprised of samples from the UK, France, Norway, Sweden, Denmark and Germany (Fig. 1). Four European sites (Rügen, Trelleborg, Hovedstaden, Fiskebäckskil), all of which are located close to each other on either side of the Danish Straits, harboured haplotypes from both the Eastern and the Western clade (Fig. 1). The two Canadian sites formed their own clades (posterior probabilities = 1), with the exception of a single individual from Baffin Island, which belonged to the most common Eastern European clade haplotype (haplotype E3). The N_ST_ distance based tree is not presented as it provided no additional information. TMRCA for the three main clades (Canadian clades are treated as one) was estimated to be 1.62 (95% HPD, 0.96–2.30) Mya. The split between the two European clades was dated at 1.48 (95% HPD, 0.80–2.20) Mya.

### Genetic diversity

**Figure 3:**
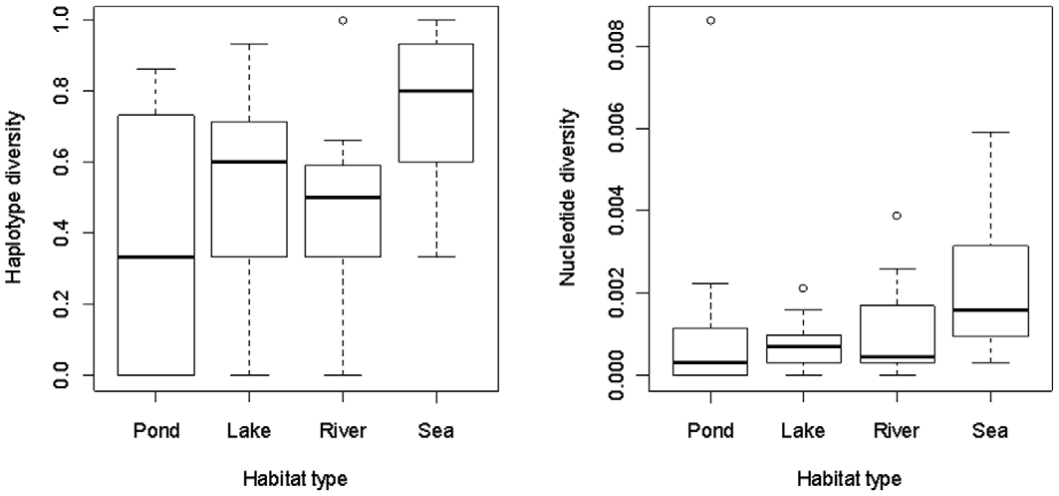
Box plots showing the distribution of haplotype and nucleotide diversity for each habitat type. Outliers are shown with open circles, the range (excluding outliers) is marked by the upper and lower horizontal lines, the main boxes denote the inter-quartile range, and the heavy central lines show the medians.

Resolution within the main clades remains low, and there was no evidence of clustering of haplotypes by habitat type (Fig. 2). Likewise N_ST_ based trees did not provide evidence for clustering within clades by habitat type (results not presented). Using the complete dataset, there was no evidence of any effect of longitude (*F*_(1,50)_ = 0.204, *p* = 0.654), or latitude (*F*_(1,50)_ = 1.847, *p* = 0.180) on haplotype diversity, but there was significant evidence for an effect of habitat type on haplotype diversity (*F*_(3,50)_ = 2.812, *p* = 0.049). Sequentially dropping terms had little effect on these results. Tukey's tests revealed that all pairwise comparisons among habitat types were non-significant, except in the case of the Sea-Pond comparison (*p* = 0.030; Mean haplotype diversities: Pond = 0.404; Lake = 0.507; River = 0.482; Sea = 0.739). When limiting the analyses to include only freshwater samples, or to include only the Western or the Eastern clade samples, all the results became non-significant. Using the complete dataset, there was no evidence that nucleotide diversity is affected by longitude (*F*_1,50_ = 0.131, *p* = 0.719), and borderline evidence that it is affected by habitat type (*F*_3,50_ = 2.404, *p* = 0.078), but there is strong evidence that nucleotide diversity declines with increasing latitude (*F*_1,50_ = 8.334, *p* = 0.006). For freshwater samples only, the results remain qualitatively the same (*F*_1,40_ = 5.483, *p* = 0.024). However, all tests become non-significant when only looking within only the Eastern or Western clades, most likely simply due to the reduction in sample size. Box plots showing the distribution of haplotype and nucleotide diversities for each habitat show that in general coastal populations exhibit higher diversities than freshwater populations, and that the ponds had the lowest median diversities among the freshwater habitat types (Fig. 3). Correlations between microsatellite heterozygosity [obtained from ref. 13] and both nucleotide and haplotype diversities across populations were positive and significant (*r_s_* = 0.529, S = 3363.30, n = 35, *p* = 0.001; *r_s_ = *0.561, n = 35, S = 3135.300, *p* = 0.0005, respectively).

## Discussion

Our data confirm a clear split between the eastern and western clades of the nine-spined stickleback in Europe, and now clarify the region where these clades meet and overlap. Interestingly, the age of these clades (ca. 1.5 Mya) appears to date back to far before the last Pleistocene glacial period (ca. 110-10 Kya), to the mid Pleistocene epoch (ca. 2.6 Mya–10 Kya), suggesting long independent evolution of the two clades. Furthermore, in accordance with microsatellite data [13], the mitochondrial nucleotide diversity decreased towards the North, as would be expected if the populations have undergone repeated bottlenecks during a northwards expansion. When looking at the different habitat types, it is clear that coastal populations had higher nucleotide and haplotype diversities than the freshwater populations, but little difference was evident between the freshwater populations. Finally, the sequences clustered by geographic proximity, rather than by habitat type. Had the samples clustered by habitat type, this would have implied that a single colonisation event had occurred from marine to freshwater, or vice versa. However as coastal and freshwater individuals were found spread throughout each clade, with no clear groupings, it is likely that the colonisation events between marine/freshwater environments occurred more than once. In the following sections, we discuss these findings and relate them to earlier findings from similar studies.

### Broad-scale phylogeography

A previous broad-scale study of three-spined sticklebacks revealed a large clade which ranges from the East coast of the USA across to continental Europe, and forms the basal clade to European lineages [51]. Recent work by Aldenhoven et al. [30] has shown similar patterns with nine-spined sticklebacks in North America probably originating from Asia, and likely dispersing throughout North America from three major refugia (Mississippi, Bering, and Atlantic), with divergence between these groups occurring a long time before the most recent glacial period. Within their study, they also found that some European samples (Sweden and Ireland) appear to have diverged from the East Coast of North America clade approximately 250,000 years ago, whilst others (Sweden and Russia) appear to have diverged later from an Alaskan clade (node not dated [30]). Our data concurs with these patterns – our Canadian clades were of a similar age to our two main European clades, and similarly to the North American clades from Aldenhoven et al.'s [30] study, have apparently diverged long before last glaciations, though our dates indicate the split to have occurred much earlier (around 1.5 million years ago, compared to their result of 250 thousand years ago for the split between North America and Europe). However, one Canadian individual had a haplotype that is found commonly in Eastern Europe, perhaps representing an ancestral haplotype. Combining these results, it seems possible that a widespread Atlantic lineage and a less extensive Eastern lineage existed in pre-glacial times, with more gene flow and the maintenance of higher genetic diversity possible in the Atlantic. Postglacial colonisations from Eastern refugia into the Baltic/White Sea, and from the West (Atlantic) to Western Europe/North Sea and North America could explain the main patterns observed; a high diversity in the Western clade, and the lower diversity in the Eastern clade. We found no evidence for a unique clade in the White Sea region, and as such, there is no evidence for a separate refugium in this region for the nine-spined stickleback, contrary to evidence from Atlantic salmon [16], [52].

### Phylogeography within Europe

Our increased sampling intensity (number of individuals) and density (number of sampling sites) – and in particular the addition of a number of samples from Norway, Sweden, and Germany – allowed us to clarify the location of the secondary contact zone between the two main European clades identified by Shikano et al. [13]. The contact zone was located roughly speaking along the Norway/Sweden border (though the range of this species does not extend to the border except in the very South), and across the Danish Straits, where four sites were found to have haplotypes belonging to both major clades. The Danish Straits have likely acted as a semi-permeable barrier to migration, allowing limited gene flow between the two clades. A similar pattern has been found in Atlantic salmon [14]. From the distribution of the two major European clades, it seems probable that the Eastern clade has penetrated towards the West using the Baltic Sea as a dispersal route, as the sites where representatives from both clades are found fall within the range of the Western clade on both sides of the Danish Straights (Fig. 1).

A loss of nucleotide diversity from south to north was identified, but this pattern was not apparent when analysing haplotype diversity. This difference in results could be because the haplotype diversity measure is subject to large sampling variance (n = 4–7 per population), whilst the nucleotide diversity measure may be less affected by this stochasticity as it integrates variance over a number of variable nucleotide sites. A loss of diversity from south to north is expected due to the broad pattern of recolonisation from south to north, as new populations at the leading edge of a recolonisation are founded from a small number of individuals (the founder effect [20]). This pattern was also detected using microsatellites in the same species [13]. We found strong correlations between microsatellite heterozygosities reported by Shikano et al. [13] and mitochondrial nucleotide and haplotype diversities in this study. We did not detect any associated loss of diversity (as measured by haplotype diversity and nucleotide diversity) from West to East (longitude), as has been shown for a number of other fish species inhabiting the Baltic Sea [15]. It is possible that this pattern is not apparent in our dataset because our sampling included also freshwater populations as well as coastal populations from White Sea.

### Differentiation among habitat types

We found evidence that the genetic diversity (as measured by haplotype diversity but not nucleotide diversity) of coastal populations significantly exceeded that of pond populations, but not that of lake or river populations. However, though not statistically significant, our data shows that coastal populations generally have higher diversity (both haplotype and nucleotide) than all types of freshwater populations (Fig. 3). This result is intuitive, as more gene flow is possible in open coastal/marine environments, and the pattern can be expected to be the strongest between the coastal populations and the smallest freshwater populations (the pond sites). This result is also in accordance with results from microsatellite analyses [13]. No significant difference in levels of diversity could be found between river, pond and lake populations, indicating on average similar levels of gene flow and isolation from the coastal environment.

We found no evidence for clustering of haplotypes by habitat type (Fig. 2). Haplotypes clustered according to geographical proximity, rather than habitat type, and all habitat types were represented within each of the major clades. These results indicate that, regardless of whether the nine-spined stickleback has a marine or freshwater origin (the origin is, as yet, unknown), there has not been a single colonisation event from one habitat type to another. This parallels results based on microsatellite evidence from both three-spined [27], and nine-spined stickleback [13] which suggest independent origins of freshwater populations. The resolution of our sampling was not high enough to determine how many times these colonisations have occurred within the Baltic region, but fine scale sampling and the utilization of sequence information from additional genes could provide additional resolution to address this question in the future.

### Conclusions

In summary, our results have helped to clarify the location of the contact zone between the Eastern and Western lineages of the nine-spined sticklebacks in northern Europe, and indicate that the Danish Straits act as a semi-permeable barrier to migration in this species. We have demonstrated that the genetic diversity in this species declines towards the North, as expected from a South to North route of postglacial colonisation. Furthermore, as the sequences cluster by geographical proximity rather than by habitat type, it appears that adaptation to different habitat types must have occurred in parallel at different locations, rather than as a single adaptation event followed by expansion events. As expected, freshwater populations show lower genetic diversity than coastal populations, but there is little difference among the different freshwater habitat types. The results of this study also demonstrate that the footprints of post-glacial colonisation process are still clearly visible in the mitochondrial genome of the nine-spined sticklebacks, and in particular, that the fish in western and eastern parts of the Fennoscandia originate from different glacial refugia with little mixing between the long-differentiated lineages.

## Supporting Information

File S1 Samples used in the study, including information on geographical location, habitat types, number of individuals (those followed by a star were included in Shikano *et al*.'s [13] study. The numbers in brackets following the stars indicate the number of samples that were taken from the previous study), haplotypes (and the number of individuals of each haplotype in brackets), haplotype diversity and nucleotide diversity, and microsatellite heterozygosity where available (based on 12 microsatellites, taken from Shikano et al. [13]).
(DOCX)
