# Supplementary material for: Phylogeography and Genetic Structuring of European Nine-Spined Sticklebacks (Pungitius pungitius)—Mitochondrial DNA Evidence
Source: PLoS One. 2011 May 11;6(5):e19476. doi: 10.1371/journal.pone.0019476 (PMC3092751; doi:10.1371/journal.pone.0019476)
Supplement: File S1 — Samples used in the study, including information on geographical location, habitat types, number of individuals (those followed by a star were included in Shikano et al.'s [13] study. The numbers in brackets following the stars indicate the number of samples that were taken from the previous study), haplotypes (and the number of individuals of each haplotype in brackets), haplotype diversity and nucleotide diversity, and microsatellite heterozygosity where available (based on 12 microsatellites, taken from Shikano et al. [13]). (DOCX) [file pone.0019476.s001.docx]

| **Site Code** | **Location** | **Co-ordinates** | **Habitat** | **n** | **Haplotypes (number of individuals)** | **n(haplotypes)** | **Haplotype diversity** | **Nucleotide diversity** | **Microsatellite Heterozygosity** |
| --- | --- | --- | --- | --- | --- | --- | --- | --- | --- |
| FI-IIV | Finland, Kuusamo | 66° 19' 10'' N, 29° 9' 59'' E | Lake | 6 | E44 (1), E45 (4), E46 (1) | 3 | 0.600 | 0.001 | - |
| FI-JOR | Finland, Joortilojärvi | 66° 48' 57'' N, 26° 34' 01'' E | Lake | 6 | E3 (4), E47 (2) | 2 | 0.533 | 0.001 | - |
| FI-KAN | Finland, Ivalampi | 66° 13' 16'' N, 29° 9' 24'' E | Lake | 6 | E48 (1), E49 (5) | 2 | 0.333 | 0.000 | - |
| FI-KEV | Finland, Kevojärvi | 69° 45' N, 27° 01' E | Lake | 6*(3) | E4 (4), E50 (1), E51 (1) | 3 | 0.600 | 0.001 | 0.182 |
| FI-POR | Finland, Porontima | 66° 12' 34" N, 29°15' 49" E | Lake | 6 | E3 (5), E52 (1) | 2 | 0.333 | 0.000 | 0.215 |
| FI-PUL | Finland, Pulmankijärvi | 69° 58' N, 27° 58' E | Lake | 6*(4) | E3 (6) | 1 | 0.000 | 0.000 | 0.221 |
| FI-RAH | Finland, Rahajärvi | 68° 45' 10" N, 27° 21' 54" E | Lake | 5*(4) | E3 (3), E5 (1), E6 (1) | 3 | 0.700 | 0.001 | 0.326 |
| FI-RIK | Finland, Riikojärvi | 68° 06' 34'' N, 23° 34' 16'' E | Lake | 5 | E53 (4), E3 (1) | 2 | 0.400 | 0.001 | - |
| FI-SAI | Finland, Saimaa | 61° 29' N, 27° 29' E | Lake | 6*(4) | E3 (2), E21 (1), E22 (1), E90 (1), E91 (1) | 5 | 0.933 | 0.002 | 0.508 |
| FI-TUO | Finland, Tuolpujärvi | 69° 33' 47" N, 28° 02' 10" E | Lake | 6 | E3 (2), E54 (2), E55 (2) | 3 | 0.800 | 0.001 | 0.182 |
| NO-ENG | Norway, Engervann | 59° 53' 49" N, 10° 32' 02" E | Lake | 6*(6) | E28 (3), E29 (1), E30 (2) | 3 | 0.733 | 0.001 | 0.602 |
| NO-ORR | Norway, Orrevatnet | 58° 44' N, 05° 31' E | Lake | 6*(3) | E28 (2), E32 (1), E83 (1), E84 (2) | 4 | 0.867 | 0.002 | 0.544 |
| NO-STO | Norway, Storvatn | 69° 40' 0'' N, 18° 58' 0'' E | Lake | 5 | E60 (1), E61 (3), E62 (1) | 3 | 0.700 | 0.001 | - |
| NO-UGE | Norway, Ugedalsvatn | 63° 57' N, 10° 25' E | Lake | 6 | E63 (6) | 1 | 0.000 | 0.000 | - |
| RU-ONE | Russia, Onega | 61° 35' N, 34° 38' E | Lake | 5*(3) | E3 (5), E20 (1) | 2 | 0.400 | 0.000 | 0.492 |
| SE-L1 | Sweden, 'Lake' | 67° 53' 34'' N, 20° 50' 32'' E | Lake | 6*(3) | E3 (3), E7 (3) | 2 | 0.600 | 0.001 | 0.277 |
| SE-SKA | Sweden, Västra-Skavtrask | 64° 25' 38" N, 19° 26' 48" E | Lake | 6 | E3 (6) | 1 | 0.000 | 0.000 | 0.155 |
| UK-BUT | Scotland, Island of Bute | 55° 48' 32'' N, 5° 05' 17'' W | Lake | 5 | E30 (1), E68 (1), E69 (3) | 3 | 0.700 | 0.001 | - |
| UK-HAR | Scotland, Harlow | 55° 45' N, 04° 25' W | Lake | 6*(3) | E34 (6) | 1 | 0.000 | 0.000 | 0.475 |
| CA-TAM | Canada, LacTémiscouata | 47° 42' 50" N, 68° 55' 00" W | Lake | 5 | E85 (2), E86 (1), E87 (1), E88 (1) | 4 | 0.900 | 0.002 | - |
| CA-BAF | Canada, Baffin Island | 62° 52' N, 67° 21' W | Pond | 6 | E3 (1), E40 (4), E41 (1) | 3 | 0.600 | 0.002 | - |
| DK-GLO | Denmark, Hovedstaden | 55° 39' 09'' N, 12° 23' 08'' E | Pond | 5 | E30 (2), E42 (2), E28 (1) | 3 | 0.800 | 0.009 | - |
| FI-KAR | Finland, Karhulampi | 66° 39' 24'' N, 26° 26' 27'' E | Pond | 6 | E3 (1), E7 (4), E24 (1) | 3 | 0.600 | 0.001 | - |
| FI-KRK | Finland, Kirkasvetinen lampi | 66° 26' 14" N, 29° 08' 08" E | Pond | 6 | E3 (6) | 1 | 0.000 | 0.000 | 0.231 |
| FI-ONK | Finland, Onkilampi | 66° 27' 09" N, 29° 03' 32" E | Pond | 6*(3) | E10 (5), E9 (1) | 2 | 0.333 | 0.000 | 0.215 |
| FI-PYO | Finland, Pyöreälampi | 66° 15' 40" N, 29° 26' 00" E | Pond | 6*(3) | E3 (6) | 1 | 0.000 | 0.000 | 0.004 |
| FI-RYT | Finland, Rytilampi | 66° 23' 03" N, 29° 19' 12" E | Pond | 6*(3) | E3 (6) | 1 | 0.000 | 0.000 | 0.178 |
| LA-ZUL | Latvia, Zulniekvalks | 57° 27' 18'' N, 22° 52' 15'' E | Pond | 6 | E3 (5), E59 (1) | 2 | 0.333 | 0.000 | - |
| NO-OXN | Norway, Øxnevadtjern | 58° 47' 57" N, 05° 40' 34" E | Pond | 6 | E28 (6) | 1 | 0.000 | 0.000 | 0.450 |
| RU-BOL | Russia, Bolotnoje | 66° 17' 43" N, 33° 21' 58" E | Pond | 6 | E3 (2), E64 (2), E65 (1), E66 (1) | 4 | 0.867 | 0.001 | 0.538 |
| RU-KRU | Russia, Krugloje | 66° 17' 53" N, 33° 20' 45" E | Pond | 6 | E3 (3), E66 (1), E67 (1), E97 (1) | 4 | 0.800 | 0.002 | 0.465 |
| RU-MAS | Russia, Mashinnoje | 66° 17' 31" N, 33° 22' 54" E | Pond | 6 | E3 (5), E66 (1) | 2 | 0.333 | 0.000 | 0.517 |
| SE-ABB | Sweden, Abbortjärnen | 64° 28' 42" N, 19° 26' 11" E | Pond | 6 | E3 (1), E19 (5) | 2 | 0.333 | 0.000 | 0.264 |
| SE-BYN | Sweden, Bynastjärnen | 64° 27' 19'' N, 19° 26' 40'' E | Pond | 6*(6) | E3 (3), E18 (1), E19 (2) | 3 | 0.733 | 0.001 | 0.130 |
| SE-HAN | Sweden, Hansmyrtjärn | 64° 33' 24" N, 19° 10' 26" E | Pond | 6 | E76 (5), E77 (1) | 2 | 0.333 | 0.001 | 0.249 |
| SE-HOH | Sweden, Gotland | 56° 57' 08'' N, 18° 12' 43'' E | Pond | 6 | E3 (3), E46 (1), E78 (1), E79 (1) | 4 | 0.800 | 0.002 | - |
| SE-NAV | Sweden, Lil-Navartjärn | 64° 33' 53" N, 19° 11' 55" E | Pond | 6 | E80 (6) | 1 | 0.000 | 0.000 | 0.052 |
| BE-LEU | Belgium, Leuwen | 50° 52' N, 04° 42' E | River | 3*(3) | E35 (1), E36 (2) | 2 | 0.667 | 0.004 | 0.493 |
| DK-GUD | Denmark, Guden | 56° 11' N, 09° 38' E | River | 7*(3) | E28 (5), E30 (1), E82 (1) | 3 | 0.524 | 0.001 | 0.460 |
| EE-PUR | Estonia, Purste | 59° 25' N, 26° 59' E | River | 6*(3) | E3 (1), E23 (1), E24 (1), E89 (1), E94 (1), E95 (1) | 6 | 1.000 | 0.003 | 0.586 |
| FR-MON | France, Montagny les Seurre | 47° 01' 27" N, 05° 15' 16" E | River | 4*(3) | E37 (3), E38 (1) | 2 | 0.500 | 0.000 | 0.481 |
| GE-KAP | Germany, Schleswig | 54° 32' 9'' N, 9° 38' 30'' E | River | 4 | E34 (3), E56 (1) | 2 | 0.500 | 0.000 | - |
| NO-FIN | Norway, Finnevatnet | 70° 10' 0'' N, 30° 19' 0'' E | River | 6*(5) | E1 (1), E2 (5) | 2 | 0.333 | 0.000 | 0.146 |
| PL-PAS | Poland, Pasleka | 54° 04' N, 19° 39' E | River | 6*(3) | E39 (5), E92 (1) | 2 | 0.333 | 0.000 | - |
| SE-LUN | Sweden, Lund | 55° 43' N, 13° 26' E | River | 6 | E28 (6) | 1 | 0.000 | 0.000 | - |
| EE-TAG | Estonia, Saarenmaa | 58° 25' 05'' N, 22° 06' 33'' E | Sea | 1 | E3 (1) | 1 | NA | NA | - |
| FI-HEL | Finland, Helsinki | 60° 12' 09" N, 25° 10' 58" E | Sea | 6*(3) | E3 (5), E43 (1) | 2 | 0.333 | 0.001 | 0.590 |
| FI-KIV | Finland, Oulu, Kiviniemi | 65° 00' N, 25° 28' E | Sea | 6*(4) | E3 (2), E13 (1), E14 (1), E15 (1), E46 (1) | 5 | 0.933 | 0.002 | 0.578 |
| FI-PYH | Finland, Pyhäjoki | 64° 29' N, 24° 13' E | Sea | 6*(4) | E3(5), E17 (1) | 2 | 0.333 | 0.000 | 0.577 |
| GE-RUG | Germany, Rügen | 54° 24' N, 13° 13' E | Sea | 6 | E3 (2), E24 (1), E28 (1), E57 (1), E58 (1) | 5 | 0.933 | 0.006 | - |
| LA-KOL | Latvia, Kolka | 57° 45' 1'' N, 22° 36' 4'' E | Sea | 3 | E3 (1), E55 (2) | 2 | 0.667 | 0.001 | - |
| PL-GDY | Poland, Gdynia | 54° 23' 50'' N, 18° 31' 42'' E | Sea | 6 | E3 (4), E24 (1), E27 (1) | 3 | 0.600 | 0.001 | - |
| RU-LEV | Russia, Levin Navolok | 66° 17' 26" N, 33° 26' 03" E | Sea | 6*(4) | E3 (3), E11 (1), E12 (1), E93 (1) | 4 | 0.800 | 0.002 | 0.531 |
| SE-BOL | Sweden, Bölesviken | 63° 39' 41" N, 20° 12' 43" E | Sea | 6 | E3 (4), E70 (1), E71 (1) | 3 | 0.600 | 0.001 | 0.602 |
| SE-FIS | Sweden, Fiskebäckskil | 58° 14' N, 11° 24' E | Sea | 6 | E3 (1), E28 (1), E72 (1), E73 (1), E74 (1), E75 (1) | 6 | 1.000 | 0.005 | - |
| SE-FOR | Sweden, Forsmark | 60° 24' 30'' N, 18° 11' 30'' E | Sea | 6*(4) | E3 (2), E25 (1), E26 (1), E27 (1), E96 (1) | 5 | 0.933 | 0.002 | 0.604 |
| SE-TRE | Sweden, Trelleborg | 55° 22' N, 13° 9' E | Sea | 5 | E3 (1), E9 (1), E28 (1), E51 (1), E81 (1) | 5 | 1.000 | 0.005 | - |
